# Progesterone Receptor Expression in Different Regions of Glioblastoma Multiforme and in an In Vitro Model

**DOI:** 10.3390/ijms27209253

**Published:** 2026-10-18

**Authors:** Donata Szubert, Klaudyna Kojder, Dariusz Jeżewski, Maciej Tarnowski, Patrycja Tomasiak, Katarzyna Piotrowska, Agnieszka Kolasa, Mateusz Bosiacki, Izabela Szućko-Kociuba, Maciej Kołodziejczak, Patrycja Kapczuk

**Affiliations:** 1 Department of Biochemistry and Medical Chemistry, Pomeranian Medical University in Szczecin, Powstańców Wlkp. 72, 70-111 Szczecin, Poland; donata.szubert@pum.edu.pl (D.S.); mateusz.bosiacki@pum.edu.pl (M.B.); maciej.kolodziejczak@pum.edu.pl (M.K.); 2 Department of Anaesthesiology and Intensive Care, Pomeranian Medical University in Szczecin, Unii Lubelskiej 1, 71-252 Szczecin, Poland; klaudyna.kojder@pum.edu.pl; 3 Department of Neurosurgery and Pediatric Neurosurgery, Pomeranian Medical University in Szczecin, Unii Lubelskiej 1, 71-252 Szczecin, Poland; dariusz.jezewski@pum.edu.pl; 4 Department of Applied Neurocognitivistics, Pomeranian Medical University in Szczecin, Unii Lubelskiej 1, 71-252 Szczecin, Poland; 5 Department of Physiology in Health Sciences, Pomeranian Medical University in Szczecin, Żołnierska 54, 70-210 Szczecin, Poland; 6 Institute of Physical Culture Sciences, University of Szczecin, 70-453 Szczecin, Poland; patrycja.tomasiak@usz.edu.pl; 7 Department of Physiology, Pomeranian Medical University in Szczecin, Powstańców Wlkp. 72, 70-111 Szczecin, Poland; katarzyna.piotrowska@pum.edu.pl; 8 Department of Histology and Embryology, Pomeranian Medical University in Szczecin, Powstańców Wlkp. 72, 70-111 Szczecin, Poland; agnieszka.kolasa@pum.edu.pl; 9 Department of Molecular Biology, Genomics and Epigenetics, Institute of Biology, University of Szczecin, 13 Wąska, 71-415 Szczecin, Poland; izabela.szucko-kociuba@usz.edu.pl; 10 The Centre for Molecular Biology and Biotechnology, University of Szczecin, 13 Wąska, 71-415 Szczecin, Poland

**Keywords:** progesterone receptor, glioblastoma multiforme, hypoxia, nutrient deficiency, nuclear sex hormone receptors

## Abstract

The progesterone receptor (PGR) is involved in processes associated with tumor progression, also in glioblastoma multiforme (GBM). GBM is characterized by regional heterogeneity, with distinct tumor areas differing in their cellular profiles and access to nutrients and oxygen. The aim of this study was to assess the effect of the tumor microenvironment on PGR mRNA and protein expression and to characterize PGR expression in different regions of GBM. To this end, GBM tumor tissues were collected from patients, and an in vitro model was established using the U87 cell line to reproduce hypoxic (H) and nutrient-deficient (ND) conditions. PGR mRNA expression was assessed by real-time PCR, while PGR protein expression was analyzed using ELISA, Western blot, confocal microscopy, and immunohistochemistry. The microenvironmental conditions examined, including H and ND, did not affect PGR protein expression in U87 cells, while H conditions significantly increased PGR mRNA expression. Different GBM regions showed variable levels of PGR protein expression, with the highest expression observed at the peritumoral area. No effect of sex on PGR expression was observed in any of the GBM regions analyzed. Overall, the results indicate regional heterogeneity of PGR expression in GBM.

## 1. Introduction

Glioblastoma multiforme (GBM) is a malignant tumor of the central nervous system (CNS). GBM accounts for approximately 52.2% of all malignant brain and CNS tumors [1]. The average annual age-adjusted incidence rate (AAAIR) of GBM is approximately 3.28 cases per 100,000 persons [1]. According to Luo et al. [2], the 3-year overall survival of patients with GBM is 13.1%. GBM is the most common malignant glioma in adults and one of the CNS tumors with the poorest prognosis; therefore, research into its broadly understood biology is an important scientific objective.

The progesterone receptor (PGR) belongs to the nuclear receptor family, nuclear receptor subfamily 3, group C, member 3 (NR3C3). Inactive PGR occurs in protein complexes located mainly in the cytoplasm and cell nucleus. After progesterone (P) binds through the ligand-binding domain, the receptor undergoes dimerization and activation [3,4]. Activated PGR binds to progesterone response elements (PREs) in the promoter or distal enhancer of the target gene [5,6]. *PGR* transcription is regulated by several transcription factors. In human glioblastoma cells, CCAAT enhancer binding protein beta (C/EBPβ), Sp1 transcription factor (Sp1), and estrogen receptor alpha (ERα) have been shown to bind the progesterone receptor B (PR-B) promoter. C/EBPβ negatively regulates PR-B expression, whereas estradiol reduces C/EBPβ binding to the promoter and increases PR-B expression, indicating the involvement of ERα-dependent mechanisms in PGR transcriptional regulation [7]. PGR undergoes various post-translational modifications, such as phosphorylation, ubiquitination, acetylation, and methylation. These processes affect the level of PGR expression, its transcriptional activity, and the selection of target genes whose transcription is stimulated by PGR [8]. Lysophosphatidic acid (LPA), whose activity has been demonstrated in many cancers, has been shown to induce the activation of protein kinase C (PKC). PKC phosphorylates PGR, thereby increasing its transcriptional activity [9]. In turn, the transcriptional activity of PGR leads to increased *VEGF* expression [9]. This mechanism was confirmed by measuring increased *VEGF* expression after treating GBM cells with LPA [9]. In the absence of ligand, PGR may also be phosphorylated by mitogenic protein kinases, such as CDK2 (cyclin-dependent kinase 2), CK2 (casein kinase 2), and MAPK [10,11]. PGR phosphorylation occurs in response to local growth factors or in a cell cycle-dependent manner. In a highly mitogenic environment, such as that found in tumors, PGR may be activated in the absence of its ligand, progesterone. PGR may also influence gene transcription indirectly by binding to other transcription factors, such as AP-1, SP1, NF-kB, and STAT3 [5]. PGR also exerts non-genomic effects through activation of the SRC family kinase cascade [12]. PGR may also affect gene transcription through its involvement in the local and global three-dimensional organization of chromatin [13].

P has been shown to induce the invasion and migration of human astrocytoma cells (U373) [14] and GBM cells (D54, U251, and U87) [15,16]. A PGR antagonist abolished these effects [14,15]. Examination of the effect of the PGR antagonist RU486 (mifepristone) demonstrated that P affects cell growth through the classical genomic mechanism [17]. The involvement of P/PGR signaling in these mechanisms was confirmed by assessing the expression and effect of progesterone-induced blocking factor (PIBF), a PGR target gene [16]. PIBF has been shown to be involved in the IL-4Rα/JAK1/STAT6 pathway [18]. In other tumor models, PIBF has been shown to induce EGF production, thereby promoting the transcription of matrix metalloproteinase (*MMP*) genes and, consequently, cell invasion and migration [19,20]. PIBF expression has also been shown to increase after cells were exposed to P, while the use of RU486 blocked the hormone’s inductive effect [16]. Progesterone may also inhibit the proliferation and migration of human glioma cells; however, this effect occurs at non-physiologically high concentrations. At lower concentrations, the effect is consistent with that observed in the studies discussed above [21]. It has been demonstrated that combining high doses of P with temozolomide further enhanced the inhibition of GBM cell proliferation and migration [22]. The proliferation and migration of GBM cells were also affected by a P metabolite produced in the brain, 5-alpha-dihydroprogesterone (5αDHP; 5α-dihydroprogesterone), which also acts through PGR [23]. Another P metabolite formed from 5α-DHP, allopregnanolone (3α-THP; allopregnanolone), also increased the proliferation of human glioma cells (U87) and promoted the expression of genes important in GBM development: *TGF-β1*, *EGFR*, *VEGF*, and *cyclin D1* [24]. It has also been shown that progesterone (P) induced the proliferation of grade III and IV human astrocytoma cell lines through PGR [17]. P stimulates proliferation in the breast epithelium and is considered a risk factor for breast cancer. The role of progesterone and its receptor is widely examined in endometrial [25], prostate [26], and thyroid cancers [27].

GBM is characterized by substantial cellular heterogeneity within the tumor [28]. The tumor consists of various neoplastic cell lineages [29], blood vessels, diverse infiltrating and resident immune cells [30], and cancer stem cells (CSCs) [31]. Massive growth and abnormal angiogenesis result in variable access to nutrients and oxygen, creating microenvironmental heterogeneity [30]. These variables lead to the formation of different tumor niches within a single tumor, such as the hypoxic, non-proliferative tumor core; the region of intense tumor growth occurring around blood vessels; and the tumor-margin region, which strongly infiltrates the surrounding CNS tissue [30].

Data from other hormone-dependent cancers suggest that PGR expression may increase under hypoxic conditions, including through ERα-dependent mechanisms [32]. *PGR* transcription is regulated by ERα. ERα binds to estrogen response elements (EREs) located in both proximal and distant regulatory regions of the *PGR* gene, contributing to estrogen-dependent regulation of its transcription [32,33]. In our previous study, we observed higher ERα (*ESR1*) mRNA expression under hypoxic conditions in a cell model [34]. However, this relationship has not yet been clearly confirmed in GBM.

Male sex in patients with GBM is associated with higher incidence [1] and lower survival [35,36,37], and attempts to molecularly characterize GBM by sex represent an important and promising research objective [38].

The more favorable GBM incidence and survival rates observed in females, together with higher P levels in women, could indicate a potential protective role of P/PGR signaling. However, given the possibility of progesterone-independent PGR activation, its effect on glial cell proliferation, and the observed higher expression in tumors of higher malignancy grade, the available data suggest that P/PGR signaling may promote GBM progression. At the same time, its precise biological role remains unclear. The environmental effects of individual tumor-niche regions appear to be an important variable inducing PGR activation. It therefore seems justified to examine the effect of individual variables present within the tumor on PGR expression and to determine differences in PGR expression across individual regions of the GBM tumor.

## 2. Results

### 2.1. Progesterone Receptor Expression in the In Vitro Model Under Conditions of Nutrient Deficiency and Hypoxia

**Figure 1:**
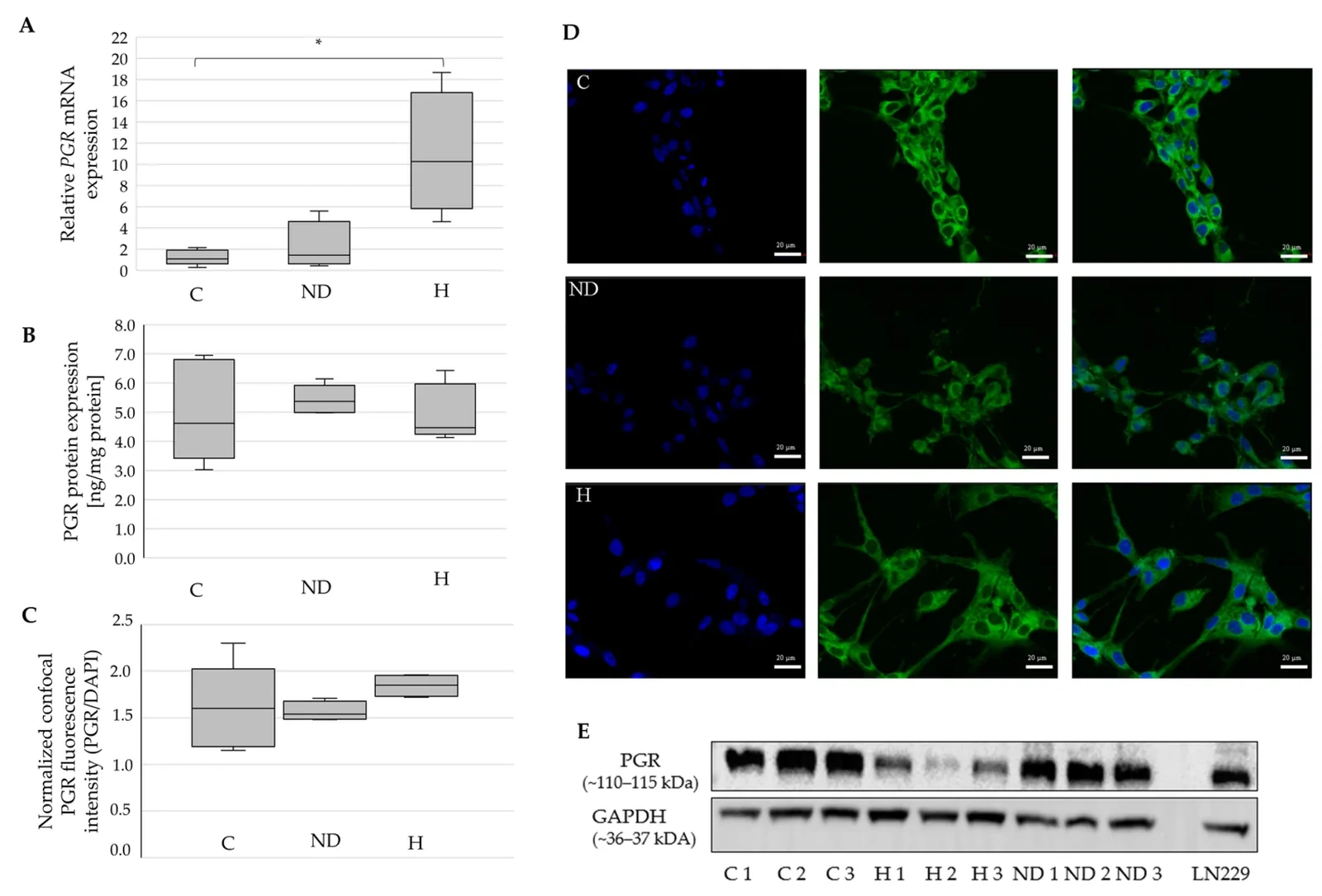
*PGR* gene (**A**) and PGR protein (**B**–**E**) expression in U87 cells cultured under different conditions. Data are shown for cells cultured under control (**C**), nutrient-deficient (ND), and hypoxic (H) conditions. Relative *PGR* mRNA expression was calculated using the 2−^ΔΔCt^ method and normalized to RPL13A and RACK1. PGR protein levels were determined by ELISA (**B**). Quantitative analysis of confocal microscopy images is shown in panel (**C**). Total PGR fluorescence intensity was normalized to total DAPI fluorescence intensity and expressed as the PGR/DAPI ratio. Panel D presents representative confocal images obtained with an FV1000 system (Olympus, Hamburg, Germany). PGR immunofluorescence was visualized with FITC, while nuclei were counterstained with DAPI. Images for the control and nutrient-deficient groups were acquired at ×20 magnification, whereas images from the hypoxic group were acquired at ×40 magnification. The scale bar represents 20 µm. Representative Western blot images of PGR and GAPDH are shown in panel (**E**). Sample labels C1–C3, H1–H3, and ND1–ND3 indicate individual samples obtained under control, hypoxic, and nutrient-deficient conditions, respectively. Statistical analysis was performed using *t*-test, * *p* < 0.05. The LN229 cell line was used as a reference, as PGR expression had previously been confirmed in these cells.

Analysis of *PGR* mRNA expression revealed a significant increase in relative expression levels in U87 cells cultured under hypoxic conditions (H) compared with control cells (C) (*p* = 0.007) (Figure 1A). In contrast, nutrient deprivation (ND) did not significantly affect *PGR* expression relative to the control.

PGR protein levels determined by ELISA did not differ significantly between the experimental conditions (Figure 1B). The mean PGR protein expression level for all experimental conditions was 5 ng/mg total protein.

Quantitative analysis of confocal microscopy images also showed no significant differences in normalized PGR fluorescence intensity between the control, hypoxic, and nutrient-deficient conditions (Figure 1C). PGR fluorescence was normalized to DAPI fluorescence and expressed as the PGR/DAPI ratio. PGR immunofluorescence was detected in all experimental conditions, with staining observed in both the cytoplasmic and nuclear regions of the cells (Figure 1D).

Western blot analysis confirmed the presence of PGR protein in U87 cells under all experimental conditions (Figure 1E). PGR-immunoreactive bands were detected in control, hypoxic, and nutrient-deficient samples, as well as in the reference LN229 cell line. A visually lower PGR band intensity was observed in samples exposed to hypoxic conditions compared with control samples, although this difference was not quantitatively assessed.

### 2.2. Progesterone Receptor Expression in the Non-Proliferative Core, Enhancing Tumor Region, and Peritumoral Area of Glioblastoma Multiforme

**Figure 2:**
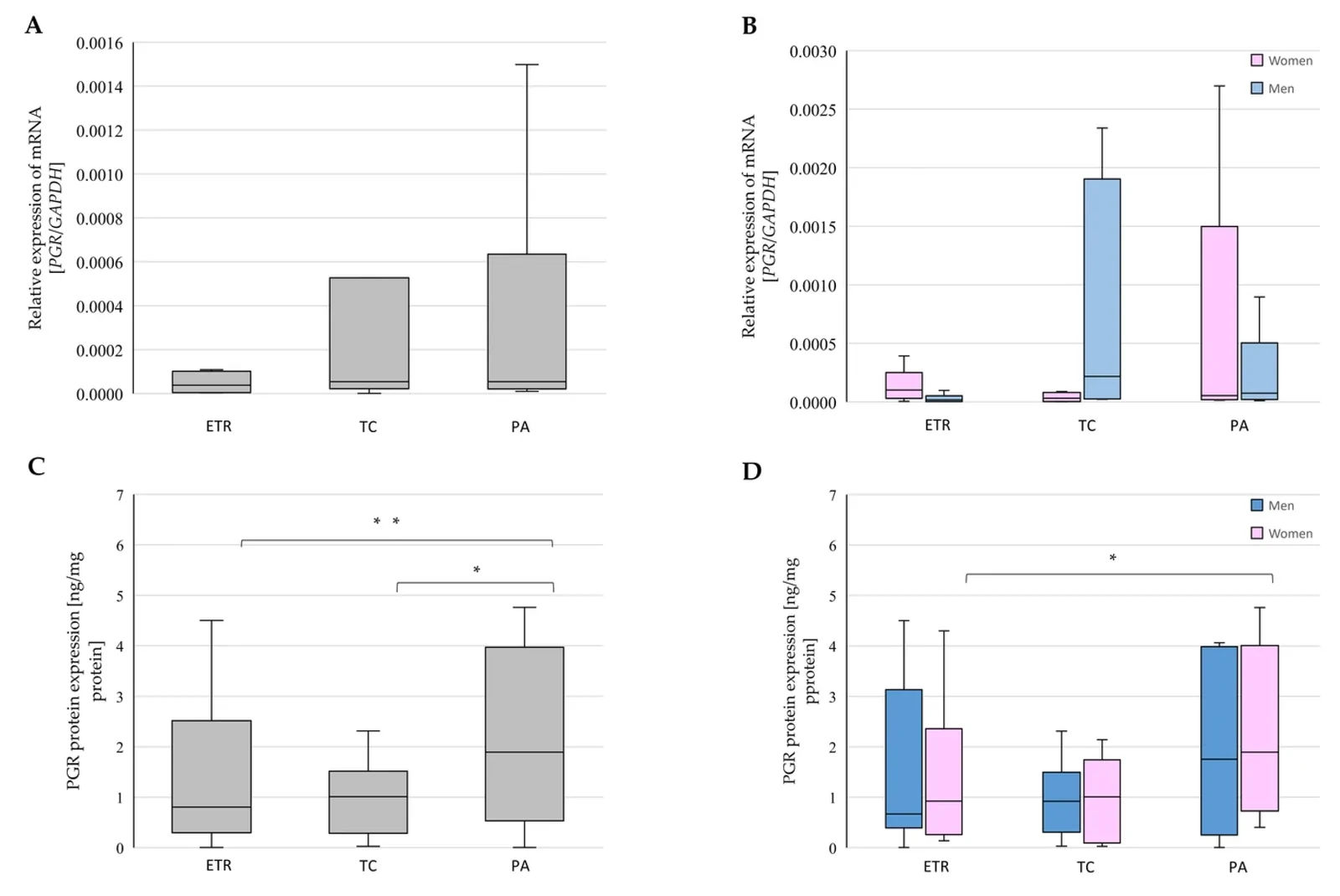
Regional distribution of *PGR* mRNA (**A**,**B**) and protein (**C**,**D**) in GBM tissue samples. Results are presented for the tumor core (TC), enhancing tumor region (ETR), and peritumoral area (PA), either for the whole study group (**A**,**C**) or separately by sex (**B**,**D**). For PGR mRNA analysis, the number of samples was TC, *n* = 11; ETR, *n* = 11; and PA, *n* = 17. For PGR protein analysis, the number of samples was TC, *n* = 17; ETR, *n* = 13; and PA, *n* = 20. Regional comparisons of PGR protein expression were performed using paired samples available for each comparison: TC vs. ETR, *n* = 12; TC vs. PA, *n* = 15; and ETR vs. PA, *n* = 12. Regional differences were evaluated using the Wilcoxon signed-rank test; * *p* < 0.05,** *p* < 0.01.

*PGR* gene mRNA expression did not differ between tumor structures in either women or men, or between sexes within the same structure (Figure 2A,B). The mean *PGR* mRNA expression level was 0.00031 [*PGR/GAPDH*].

Before sex was included in the analysis, PGR protein expression was significantly higher in the peritumoral area than in the tumor core (*p* < 0.05) or the enhancing tumor region (*p* < 0.01). PGR protein expression was higher by 210% and 144%, respectively (Figure 2C).

After sex was included in the analysis, a statistically significant difference was found only between the enhancing tumor region and the peritumoral area, with PGR expression again higher, by 161%, in the peritumoral area in women (*p* < 0.05) (Figure 2D).

**Figure 3:**
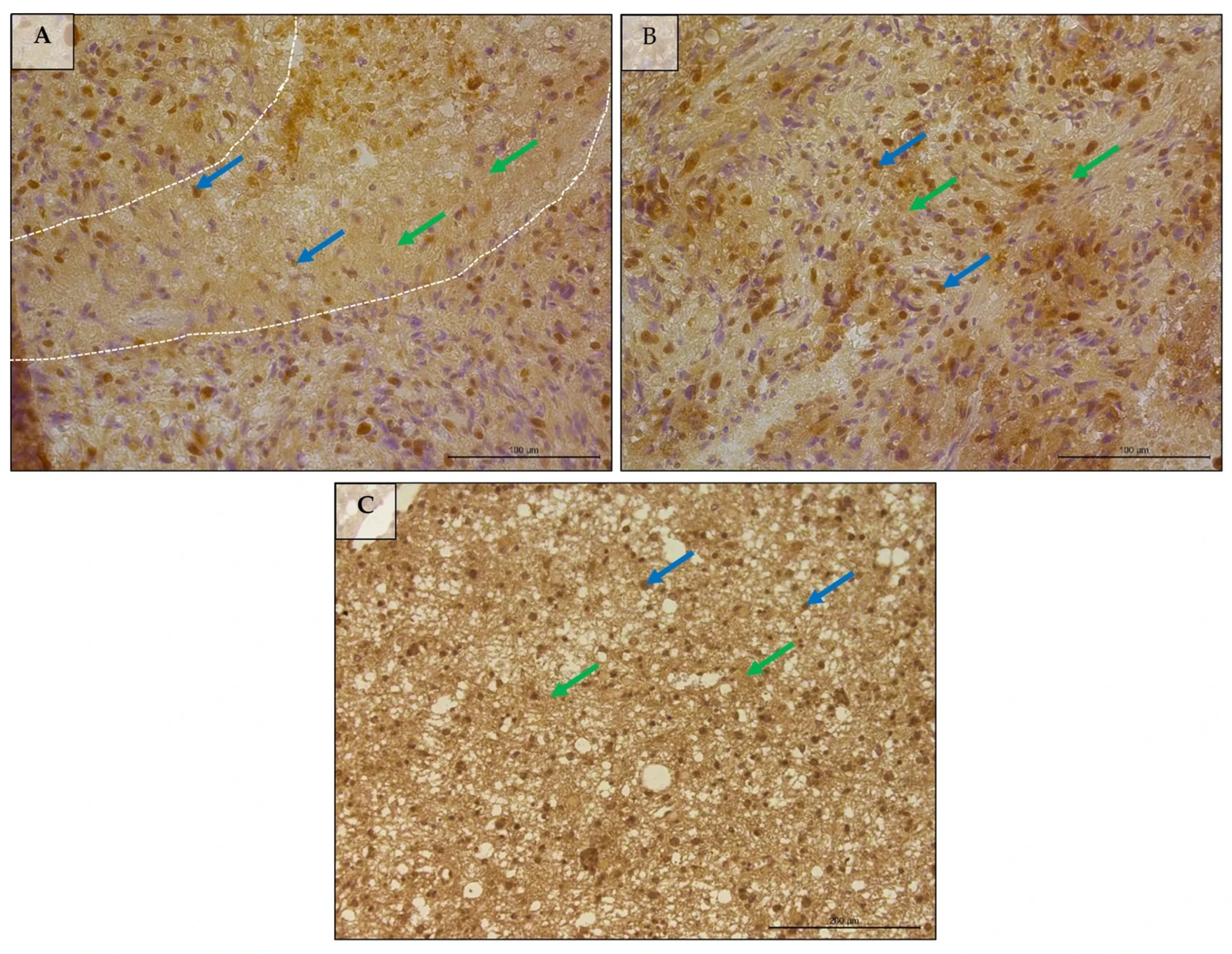
Representative microphotographs from a female patient show PGR protein expression in the tumor core, with the necrotic area circled with a white dotted line (**A**); the enhancing tumor region (**B**); and the peritumoral area (**C**) of a tumor diagnosed as GBM. A positive IHC reaction is indicated by arrows: blue—positive nuclear reaction; green—positive cytoplasmic reaction. Microphotographs were taken at ×40 magnification; scale bar: 100 µm in panels A and B and 200 µm in panel C. PGR-positive cells were quantified using QuPath software(version 0.7.0, University of Edinburgh, Edinburgh, UK).

Immunohistochemical analysis showed PGR protein expression in all three structures examined (Figure 3). Quantitative analysis of the representative case shown in Figure 3 using QuPath showed that the percentage of PGR-positive cells was highest in the peritumoral area (78.63%), followed by the enhancing tumor region (59.19%) and the tumor core (52.30%). In line with the ELISA results, PGR protein expression was highest in the peritumoral area, which showed a high degree of both cytoplasmic and nuclear staining (Figure 3C, green and blue arrows). Expression in the enhancing tumor region showed a degree of cytoplasmic staining comparable to that in the peritumoral area. However, it was characterized by a lower degree of nuclear staining (Figure 3B, blue arrows). In the tumor core, PGR protein expression was mainly cytoplasmic, and the degree of cytoplasmic staining was similar to that observed in the enhancing tumor region. Nuclear staining, however, was lower (Figure 3A, green and blue arrows).

## 3. Discussion

PGR activity has been linked to processes involved in glioma progression. Hernández-Hernández et al. showed that activation of PGR by progesterone increased VEGF and EGFR expression in D54 cells and cyclin D1 expression in U373 cells, whereas treatment with a PGR antagonist inhibited these effects [39]. In addition, Piña-Medina et al. demonstrated that progesterone enhanced the migration and invasion of D54 and U251 glioma cells, while inhibition of PGR activity or expression attenuated these effects [15]. Particularly relevant to the U87 cell line used in the present study are the findings of Germán-Castelán et al., who showed in a xenograft model that PGR silencing inhibited the progesterone-induced increase in tumor size and infiltration [40].

This study examines the effect of selected tumor microenvironmental conditions on PGR expression in the U87 cell line. We found that hypoxia increases *PGR* mRNA expression in U87 cells.

These findings indicate that regulation of PGR expression may be relevant to the response of GBM cells to progesterone and modulate processes associated with tumor progression. However, an increase in *PGR* mRNA levels alone does not demonstrate increased receptor activity or a specific biological effect.

The increase in *PGR* mRNA expression observed under hypoxic conditions in the present study may be related to ERα-mediated regulation of *PGR* transcription. In our previous study performed on the same U87 cell model, ERα was detected under both control and hypoxic conditions, while ERα (ESR1) mRNA expression increased under hypoxia [34]. Park and Lee demonstrated that hypoxia can induce ligand-independent phosphorylation and transcriptional activation of ERα, including phosphorylation at Ser118 through the MAPK/ERK1/2 pathway [41]. Therefore, increased ERα transcription together with potential hypoxia-induced activation of the receptor may represent a mechanism contributing to the increased *PGR* mRNA expression observed in the present study. However, ERα phosphorylation or transcriptional activity was not assessed in our model, and this mechanism therefore remains hypothetical.

A similar finding was reported by Habib et al., who demonstrated increased *PGR* mRNA expression in rat cortical astrocytes exposed to hypoxic conditions [42]. However, substantial differences between these experimental models preclude direct extrapolation of these findings. Our results therefore provide a rationale for further investigation of the effects of hypoxia on *PGR* mRNA expression in GBM.

This observation is particularly interesting in light of the opposite effect of hypoxia on *PGR* mRNA expression reported in breast cancer cells. Previous studies have shown that hypoxia reduces *PGR* mRNA expression in MCF-7 breast cancer cells cultured both with and without estradiol stimulation [43,44].

At the same time, published data on the presence of *PGR* transcripts in U87 cells are inconsistent. The study by Piña-Medina et al. [45] reports *PGR* mRNA expression, whereas the study by Hlavaty et al. [46] shows the absence of this expression. It should be noted that *PGR* mRNA expression in the study by Piña-Medina et al. [45] was very low, almost at the limit of detection. In the study by Hlavaty et al. [46], RT-PCR was used to measure PGR mRNA; this method is generally considered less sensitive than RT-qPCR, which is quantitative and was used by Piña-Medina et al. [45].

This study provides additional independent evidence supporting *PGR* mRNA expression in U87 cells and highlights methodological approaches that may facilitate the design of future experiments investigating *PGR* transcript levels in this cell line. The difficulty in detecting *PGR* mRNA in U87 cells is particularly relevant given the existing evidence of PGR expression at the protein level.

The study also examined PGR protein expression in the U87 cell line. For this purpose, three methods were used: ELISA, Western blotting, and confocal microscopy. The use of three complementary approaches confirmed the presence of PGR protein in U87 cells. Quantitative analysis by ELISA and confocal microscopy showed no significant differences in PGR protein levels between the experimental conditions. Confocal microscopy also showed predominantly cytoplasmic PGR localization, with no clear increase in nuclear accumulation under the conditions examined. However, subcellular localization alone does not allow conclusions to be drawn regarding receptor activity. Western blotting also confirmed the presence of PGR in all analyzed conditions, although a visually lower PGR signal was observed under hypoxic conditions. As Western blotting was used primarily as a confirmatory method, this observation was interpreted qualitatively. A similar observation was reported by Habib et al. [42], who found no differences in PGR protein expression in rat cortical astrocytes exposed to normoxic and hypoxic conditions [42]. However, it should be emphasized that there are important differences between that model and U87 cells.

PGR expression in the U87 cell line remains controversial in the literature. Studies by Germán-Castelán et al. [40] and Piña-Medina et al. [45] confirm PGR protein expression, whereas Hlavaty et al. [46] did not detect nuclear PGR in U87 cells at either the mRNA or protein level and interpreted the observed progesterone effects in this model as being associated with membrane progesterone receptors. The most recent data on PGR expression in U87 cells were presented by Arcos-Montoya et al. [47], who also confirmed the presence of PGR in U87 cells. These discrepancies provided a rationale for independently confirming PGR protein expression in our U87 model using Western blotting.

The visually lower PGR signal observed by Western blot under hypoxic conditions was not evident in either the ELISA or quantitative confocal microscopy analyses, and therefore caution should be exercised when interpreting it as evidence of a reduction in total cellular PGR. It has been shown that post-translational modifications can alter antibody recognition of an epitope and consequently affect immunoblot signal intensity without a corresponding change in protein abundance [48]. Hypoxia can induce changes in protein phosphorylation [49,50,51], whereas PGR undergoes multiple post-translational modifications, particularly phosphorylation, which influence its stability, turnover, and transcriptional activity [52]. Since numerous PGR phosphorylation sites are located within the N-terminal region of the receptor recognized by the polyclonal antibody used in the present study, hypoxia-related changes in the post-translational state of PGR may have contributed to the weaker Western blot signal without a corresponding change detected by the other methods. However, this mechanism was not assessed in the present study and therefore remains hypothetical.

Large analyses of cancer cell lines have shown that mRNA levels only partially reflect the levels of the corresponding proteins, which may result from differences in translation as well as mRNA and protein stability and turnover [53,54]. A similar finding was reported directly in GBM models including the U87MG cell line, where changes induced by 24 h of hypoxia at the transcript level showed only a weak association with changes observed at the protein level [55]. In the case of PGR, the relationship between transcript and protein levels may also be complex, and discrepancies between *PGR* mRNA expression and PGR protein detection have been reported in different tumor cell lines [56]. The results obtained in the present study, in which hypoxia increased *PGR* mRNA expression without a corresponding significant increase in protein levels, are therefore consistent with the possibility of multilevel regulation of PGR in GBM cells.

It should be noted, however, that the results obtained may be characteristic of the cell line cultured in our laboratory and may not correspond to the commercially available line. This results from culture variability associated with passaging, as discussed by Hlavaty et al. [46]. The report by Piña-Medina et al. [45] showing an increase in PGR protein expression in U87 cells exposed to neural stem cell medium is also important. On this basis, it may be assumed that the results for PGR protein expression in the U87 cell line may depend on culture conditions and the resulting cellular phenotype.

Some studies have also examined this receptor functionally. Although they do not directly demonstrate receptor expression in the U87 cell line, obtaining an effect characterized as PGR-mediated may provide indirect evidence of its expression in this line. Germán-Castelán et al. [40] showed that phosphorothioated antisense oligodeoxynucleotides administered to silence *PGR* expression blocked the effects of progesterone, namely the growth and migration of U87 cells implanted into rat brains. Blocking PGR with its antagonist RU486 abolished the effects of migration and invasion obtained through PGR activation by PKC-catalyzed phosphorylation [57].

However, the functional status of PGR under the experimental conditions used in the present study was not directly assessed. This should be addressed in future studies by evaluating receptor phosphorylation and the expression of PGR target genes.

The literature data support the assumption that P/PGR signaling plays an important role in the processes of GBM growth and development. P/PGR signaling is a valuable research target in GBM, and GBM cell models are useful tools. Therefore, there is a justified need to clearly define the U87 cell line in terms of PGR expression at both the mRNA and protein levels.

The results obtained in the present study do not allow definitive conclusions to be drawn regarding the role of the microenvironment in GBM development, which was the original aim of the experiments. However, they indicate that hypoxia may regulate *PGR* expression at the mRNA level, while no significant differences in PGR protein levels were detected between the analyzed conditions. These findings provide another observation consistent with the presence of PGR protein in U87 cells.

The discrepancies between PGR mRNA and protein expression observed in cell models should also be interpreted in the context of data obtained directly from GBM tissues. Discordance between mRNA and protein expression is frequently reported in GBM [58,59]. A similar pattern has been observed for PGR, with protein expression increasing with tumor grade despite a decrease in *PGR* mRNA levels in higher-grade tumors [60].

Progesterone receptor (PGR/PR) expression has been demonstrated both in low-grade gliomas and in glioblastoma multiforme (GBM) [61,62,63]. However, several studies have observed higher receptor expression in high-grade gliomas. Carroll et al. [61] showed more frequent occurrence of *PGR* mRNA in high-grade tumors, whereas Khalid et al. [62] and Assimakopoulou et al. [63] reported a higher percentage of PGR-positive cells in GBM and anaplastic astrocytomas than in low-grade gliomas, suggesting an association between PGR expression and tumor progression [61,62,63]. In addition, Khalid et al. [62] demonstrated a correlation between PGR expression and the proliferative activity of tumor cells, indicating the potential involvement of the receptor in glioma development [62].

Subsequent studies confirmed frequent PGR expression in GBM, while also drawing attention to the predominance of the PGR-B isoform in high-grade tumors [64]. However, not all later analyses confirmed an association between PGR expression and tumor grade, as both Mezmezian et al. [65] and Tavares et al. [66] observed the presence of the receptor in gliomas regardless of grade, with no statistically significant differences between the analyzed groups. Despite these discrepancies, most available data indicate that progesterone signaling may participate in glioma biology, particularly in high-grade tumors.

In the analyses presented in this study, the examined GBM tumor tissues expressed PGR mRNA and protein. No differences in *PGR* mRNA expression were observed in relation to tumor structure or sex. The low level of *PGR* mRNA expression detected in GBM, together with the limited number of samples analyzed, may have reduced the ability to detect differences between the examined groups. This may also be explained by the observation described above that *PGR* mRNA expression is lower in malignant types of glioma, which include GBM.

In the present analyses, in contrast to the low levels of *PGR* mRNA expression, PGR protein was readily detectable in the analyzed tissues. According to our analyses, PGR protein levels were lowest in the tumor core and increased progressively in the enhancing tumor region and the peritumoral area. These observations were supported by immunohistochemical analysis, which showed a similar regional pattern, with a higher percentage of PGR-positive cells in the peritumoral area in the representative case analyzed quantitatively. This regional distribution may be biologically relevant, as the enhancing tumor region is associated with active tumor growth, whereas the peritumoral area contains infiltrating GBM cells, in contrast to the hypoxic and necrotic features of the tumor core [67,68,69]. Given the previously described involvement of PGR in GBM cell proliferation, migration, and invasion, higher PGR protein expression in the enhancing tumor region and peritumoral area may be associated with processes contributing to tumor growth and infiltration [15,40,57]. However, the present study did not directly assess the relationship between PGR expression and proliferative or invasive activity in individual tumor regions. The potential role of PGR in tumor growth has also been examined in in vivo GBM models. P-induced xenotransplantation of human U87 glioma cells into the rat cerebral cortex showed induction of tumor growth and infiltration through interaction with intracellular PGR [40]. Studies conducted in an orthotopic mouse model of GBM (U118MG) did not show tumor growth following the administration of low doses of P [70].

In the analyses presented here, after sex was included, the difference in expression was reproduced only between the enhancing tumor region and the peritumoral area, and only in women. This may suggest a possible sex-related difference in the regional pattern of PGR expression. However, no direct difference in PGR expression between women and men was observed. In light of the dose-dependent differences in the response of GBM cells to P induction, it seems important to examine differences in tumor-cell responses to P concentrations occurring in both men and women under different physiological conditions.

The results presented in this study confirm the presence of PGR in GBM and indicate regional variation in PGR protein expression within the tumor. The observed lack of correspondence between PGR mRNA and protein levels in patient tissues suggests the possible involvement of post-transcriptional or post-translational regulatory mechanisms affecting protein levels independently of transcript abundance. Higher PGR protein expression in the enhancing tumor region and peritumoral area than in the tumor core may indicate the potential involvement of progesterone signaling in processes related to the proliferation and invasiveness of tumor cells. Additionally, these tissue findings should be considered together with the effects of selected tumor microenvironmental conditions observed in the U87 model. Hypoxia increased *PGR* mRNA expression in U87 cells, whereas nutrient deprivation did not induce significant changes. At the protein level, however, neither condition significantly affected PGR levels. Therefore, the increase in *PGR* mRNA observed under hypoxic conditions in vitro does not directly contradict the lower PGR protein level observed in the tumor core, as these findings concern different levels of PGR regulation and different experimental systems.

However, it should be emphasized that the interpretation of these findings may be influenced by the experimental design. Given the persistent discrepancies in the literature regarding PGR expression in GBM models and the limited understanding of the mechanisms regulating this receptor, further studies are warranted. These should include a detailed characterization of PGR expression at both the mRNA and protein levels, as well as an assessment of the functional activity of the PGR signaling pathway under tumor microenvironmental conditions.

## 4. Materials and Methods

In the present study, we used a research design developed on the basis of a previously published study by Simińska et al. on androgen receptor expression in different regions of resected glioblastoma multiforme tumors and in an in vitro model [69]. The same experimental design was subsequently used in another study on estrogen receptor expression, which represented a continuation of the adopted methodology [34]. The present study is based on the experimental experience and methodological solutions developed in both of these studies.

### 4.1. In Vitro Model: Cell Culture

The study used an in vitro model based on the human glioblastoma cell line U87, also known as U-87 MG (European Collection of Authenticated Cell Cultures, ECACC, Salisbury, UK), to assess the effect of tumor microenvironmental conditions on the expression of the receptor examined. The experimental design was based on the methodology described in previous publications by the research team [34,69].

Cells were cultured at 37 °C, 95% humidity, and 5% CO_2_ in 6-well culture plates (Nest Biotechnology Co., Ltd., Wuxi, China) at a density of 20,000 cells/cm^2^ until 70–80% confluence was reached, in EMEM supplemented with 10% FBS, 100 U/mL penicillin, 100 µg/mL streptomycin, and 1% non-essential amino acids (EMEM, Sigma-Aldrich, St. Louis, MO, USA; FBS, Gibco, Thermo Fisher Scientific, Waltham, MA, USA; penicillin and streptomycin, Gibco, Thermo Fisher Scientific, Waltham, MA, USA; non-essential amino acids, Sigma-Aldrich, St. Louis, MO, USA).

Cells were then cultured for 24 h under control or experimental conditions reflecting selected features of the glioma microenvironment. The hypoxia model was obtained by adding 100 µM cobalt chloride (Sigma-Aldrich, St. Louis, MO, USA), nutrient-deficient conditions were induced by reducing the L-glutamine concentration to 0.2 mM and removing sodium pyruvate from the medium. Each experimental variant was performed in six replicates.

Material for gene and protein expression analyses was collected after detaching the cells from the substrate using a 0.25% trypsin-EDTA solution (Sigma-Aldrich, St. Louis, MO, USA). After two rounds of centrifugation and washing with PBS solution (Sigma-Aldrich, St. Louis, MO, USA), the resulting cell pellets were stored at −80 °C until further analysis. For immunofluorescence and confocal microscopy, cells were additionally cultured on slides coated with a 0.01% poly-L-lysine solution (BioReagent, Sigma-Aldrich, St. Louis, MO, USA). After incubation, the cells were washed with PBS, fixed in 4% buffered formaldehyde solution (Sigma-Aldrich, St. Louis, MO, USA) for 10 min at room temperature, washed again with PBS, and prepared for further microscopic analyses.

### 4.2. Model of Tumor Regions in Patients with GBM

Biological material was obtained from 24 patients (14 men and 10 women; mean age, 62 years) who underwent surgical resection of glioblastoma multiforme (GBM) at the Department of Neurosurgery and Pediatric Neurosurgery, Pomeranian Medical University in Szczecin. The diagnosis of GBM was established on the basis of neuroimaging examinations (magnetic resonance imaging and/or computed tomography) and verified histopathologically after surgery. Only adult patients with IDH-wild-type glioblastoma multiforme were included in the study. All participants were informed about the aims and course of the project and provided written informed consent to participate in the study. The project was approved by the Bioethics Committee of the Pomeranian Medical University in Szczecin (Resolution No. KB0012/96/14/A-1 of 9 March 2020) and was conducted in accordance with applicable national and international guidelines for research involving humans.

Tissue samples were collected during a standard neurosurgical procedure involving craniotomy and tumor resection. The location of individual tumor regions was determined using a neuronavigation system, which enabled precise collection of material from three distinct zones: the tumor core, the enhancing tumor region, and the peritumoral area. The tumor core corresponded to the central part of the neoplastic lesion and was characterized by the presence of hypoxic and necrotic areas. The enhancing tumor region represented the actively proliferating part of the tumor with high growth activity. The peritumoral area included tissue surrounding the tumor and contained individual infiltrating tumor cells migrating along Scherer’s secondary structures. Because of its biological properties, this area was used as reference tissue in the comparative analyses.

Collection of material from these regions enabled assessment of molecular differences between the individual tumor zones, including analysis of regional variability in the expression of the biological markers examined.

### 4.3. Quantitative Real-Time Polymerase Chain Reaction (qRT-PCR)—Cell Culture Samples

The relative expression level of the *PGR* gene in cell culture samples was determined by quantitative real-time PCR (qRT-PCR). First-strand cDNA was synthesized from 2 μg of DNase-treated total RNA in a final reaction volume of 20 μL using the RevertAid RT Kit (Thermo Fisher Scientific, Waltham, MA, USA), according to the manufacturer’s instructions. The resulting cDNA was diluted 10-fold with nuclease-free water and stored at −20 °C until further analysis. qRT-PCR analysis was performed using the SensiFAST™ SYBR^®^ No-ROX Kit (Meridian Bioscience, Cincinnati, OH, USA) on a CFX96 Touch Real-Time PCR Detection System (Bio-Rad, Hercules, CA, USA). The following primer sequences were used to amplify the *PGR* gene: forward, 5′-CCAGCCAGAGCCCACAATAC-3′ and reverse, 5′-ACAGCTCCCACAGGTAAGGA-3′, yielding a 110 bp amplicon. The *PGR* primers were designed using Primer-BLAST (National Center for Biotechnology Information, Bethesda, MD, USA) to amplify all known transcript variants of the gene. The following primer sequences were used for the reference genes: *RPL13A*, forward 5′-CATAGGAAGCTGGGAGCAAG-3′ and reverse 5′-GCCCTCCAATCAGTCTTCTG-3′; and *RACK1*, forward 5′-CTTCTGGAGGCAAGGATGGC-3′ and reverse 5′-CACACAGCCAGTAGCGGTTA-3′.

Cycling conditions were determined according to the manufacturer’s instructions, and optimized for the primers used. All reactions were performed in duplicate, and mean Ct values were used for subsequent calculations. Melting curve analysis was performed after amplification to verify the specificity of the PCR products. In addition, the products obtained with each primer pair were sequenced to confirm their identity. Amplification efficiencies were verified and ranged from 95% to 105% for all analyzed genes. Relative gene expression was calculated using the comparative Ct (ΔΔCt) method and normalized to the mean expression of two reference genes, *RPL13A* and *RACK1*. *RPL13A* and *RACK1* were selected based on prior validation by the laboratory performing the qRT-PCR analysis described here. *RPL13A* has previously been identified as a stable reference gene in analyses including U87MG cells and has also been used in U87MG cells exposed to hypoxic conditions [71,72]. *RACK1* was used as the second reference gene based on prior internal validation showing stable expression in U87 cells.

### 4.4. qRT-PCR—GBM Tissue Samples

The relative expression level of the *PGR* gene was determined by quantitative real-time PCR (qRT-PCR). Total RNA was isolated from tissue samples collected from different regions of glioblastoma multiforme using the RNeasy Lipid Tissue Mini Kit (Qiagen, Hilden, Germany), according to the manufacturer’s instructions. The concentration and purity of the RNA obtained were assessed spectrophotometrically using a NanoDrop ND-1000 instrument (Thermo Fisher Scientific, Waltham, MA, USA).

Complementary DNA (cDNA) was then synthesized by reverse transcription using the First Strand cDNA Synthesis Kit and oligo(dT) primers (Thermo Fisher Scientific, Waltham, MA, USA). qRT-PCR analysis was performed using Power SYBR Green PCR Master Mix (Applied Biosystems, Thermo Fisher Scientific, Waltham, MA, USA) on an ABI 7500 Real-Time PCR System (Applied Biosystems, Thermo Fisher Scientific, Waltham, MA, USA).

The following primer sequences were used to amplify the *PGR* gene: ACCCGCCCTATCTCAACTACC|AGGACACCATAATGACAGCCT. The reaction conditions included initial denaturation at 95 °C for 15 s, followed by 40 cycles consisting of denaturation at 95 °C for 15 s and primer annealing and elongation at 60 °C for 60 s. Each sample was analyzed in two technical replicates, and mean Ct values were used for further calculations.

*PGR* gene expression was normalized to the expression of the reference gene *GAPDH* (glyceraldehyde-3-phosphate dehydrogenase), which served as the endogenous control [73,74]. Relative expression levels were calculated on the basis of Ct values obtained for the analyzed samples from individual GBM tumor regions.

### 4.5. Enzyme-Linked Immunosorbent Assay (ELISA)

PGR protein levels were determined by ELISA in both study models, i.e., in GBM tissue samples collected from patients and in material obtained from U87 cell culture, using the Human PGR ELISA Kit (EH1449) (FineTest, Wuhan, China). To prepare samples for protein analyses, tissue fragments of approximately 1 cm^3^ were homogenized mechanically in 1 mL of chilled PBS (0.01 M, pH 7.4; Sigma-Aldrich, Poznań, Poland), whereas cell pellets containing 2 × 10^6^ cells were homogenized in 0.5 mL of RIPA buffer (Thermo Fisher Scientific, Waltham, MA, USA). Protease inhibitors were added to all homogenates (PhosSTOP and cOmplete Mini Protease Inhibitor Cocktail; Sigma-Aldrich, Poznań, Poland). Sample preparation was carried out at approximately 4 °C. Tissue homogenates were subjected to sonication, whereas cell homogenates underwent freeze–thaw cycles according to the manufacturer’s instructions. Absorbance was read using a Biochrom Asys UVM 340 microplate reader (Biochrom, Cambridge, UK), and PGR concentrations were calculated from the standard curve and normalized to the total protein content in the sample.

### 4.6. Western Blot

Western blot analysis was performed on three representative samples from each experimental condition and was used to confirm the presence of PGR protein. Protein samples were mixed with Laemmli loading buffer (Bio-Rad, Feldkirchen, Germany) containing 2-mercaptoethanol (Sigma-Aldrich, St. Louis, MO, USA) and denatured at 90 °C for 5 min in a thermomixer (Eppendorf SE, Hamburg, Germany). Proteins were separated by SDS-PAGE using an 8–16% gradient Bis-Tris SurePAGE™ polyacrylamide gel (cat. no. M00660; GenScript, Piscataway, NJ, USA), with 15 μg of protein loaded per well. Electrophoresis was performed in Tris–MOPS–SDS running buffer (cat. no. M00138; GenScript, Piscataway, NJ, USA) at 180 V for 45 min.

Following electrophoresis, proteins were transferred by wet transfer onto a PVDF membrane with a pore size of 0.2 μm (cat. no. 88520; Thermo Fisher Scientific, Waltham, MA, USA). Transfer was performed in Tris–Bicine buffer (cat. no. M00139; GenScript, Piscataway, NJ, USA) containing 20% methanol at 70 V for 60 min. The membranes were blocked for 45 min at room temperature in 5% non-fat dry milk prepared in TBS (ZELLX GmbH, Berlin, Germany) and then incubated overnight at 4 °C with a rabbit polyclonal primary antibody against PGR diluted 1:3000 (cat. no. 25871-1-AP; Proteintech Group, Inc., Rosemont, IL, USA).

GAPDH, used as a loading control, was detected using a mouse monoclonal antibody diluted 1:20,000 (cat. no. ab8245; Abcam, Cambridge, UK). The membranes were subsequently incubated for 1 h at room temperature with a goat anti-rabbit IgG H&L antibody conjugated to horseradish peroxidase (HRP) (cat. no. ab205718; Abcam, Cambridge, UK) and a goat anti-mouse IgG H&L antibody conjugated to HRP (cat. no. ab6789; Abcam, Cambridge, UK).

Chemiluminescent signals were detected using the ECL Advance Western Blotting Detection Kit (GE Healthcare, Chicago, IL, USA), and protein bands were visualized using the ChemiDoc XRS+ system (Bio-Rad Laboratories, Inc., Hercules, CA, USA). LN229 cells (ATCC CRL-2611; American Type Culture Collection, Manassas, VA, USA) were used as a reference glioblastoma cell line in which expression of the nuclear progesterone receptor (PGR) had previously been confirmed [8].

### 4.7. Immunohistochemistry

Immunohistochemical analysis of PGR expression was performed in GBM tissue samples collected from two representative patients, one woman and one man, as a complementary method to confirm PGR detection in tissue and assess its cellular localization. Tumor fragments were fixed for 24 h in 4% formaldehyde solution, dehydrated, and embedded in paraffin blocks. Paraffin blocks were cut using a Microm HM340E microtome (Thermo Fisher Scientific, Waltham, MA, USA), and the resulting sections were mounted on poly-L-lysine-coated slides (Sigma-Aldrich, Poznań, Poland). The specimens were deparaffinized, hydrated, and subjected to heat-induced antigen retrieval (HIER) by heating twice in 10 mM citrate buffer (pH 9.0; Dako Inc., Carpinteria, CA, USA) for 4 and 3 min in a microwave oven (700 W). The specimens were then cooled, rinsed with PBS, and blocked for endogenous peroxidase activity using the Peroxidase Blocking Reagent from the Dako LSAB+ System-HRP kit (Dako Inc., Carpinteria, CA, USA) for 10 min at room temperature. Sections were incubated overnight with an anti-PGR antibody (PGR Antibody, sc-810; Santa Cruz Biotechnology, Dallas, TX, USA) at a concentration of 4 µg/mL. The immune complex was then visualized using the Dako LSAB+ System-HRP kit according to the manufacturer’s instructions. 3,3′-Diaminobenzidine (DAB) was used as the chromogen, and the specimens were counterstained with hematoxylin. Images were recorded using a Leica DM5000 B light microscope (Leica, Wetzlar, Germany) equipped with a digital camera. Three microphotographs of representative tumor regions were taken for each specimen: the necrotic area, the enhancing tumor region, and the peritumoral area. Quantitative analysis of PGR immunoreactivity was performed using QuPath software (version 0.7.0, University of Edinburgh, Edinburgh, UK). Images were analyzed as brightfield H-DAB images, and cells were detected based on hematoxylin optical density. PGR positivity was determined using the mean nuclear DAB optical density, with a single positivity threshold of 0.40 applied consistently to all analyzed regions. PGR expression was expressed as the percentage of PGR-positive cells relative to the total number of detected cells.

### 4.8. Confocal Microscopy

PGR expression in U87 cells was assessed by immunofluorescence using confocal microscopy. Fixed cell culture specimens were rinsed with PBS (Sigma-Aldrich, Poznań, Poland) and then permeabilized in 0.5% Triton X-100 solution (Sigma-Aldrich, Poznań, Poland) for 20 min. After rinsing with PBS, non-specific binding sites were blocked for 20 min at room temperature using 2.5% horse serum in PBS (Thermo Fisher Scientific, Waltham, MA, USA). The specimens were then incubated for 1 h at room temperature in a humidified chamber with an anti-PGR antibody (PGR Antibody, sc-810; Santa Cruz Biotechnology, Dallas, TX, USA). After incubation, the specimens were washed with PBS and incubated for 1 h at room temperature in the dark with a FITC-conjugated secondary antibody (Merck Millipore, Poznań, Poland). The specimens were then rinsed again with PBS and incubated with DAPI (Merck Millipore, Poznań, Poland) for 20 min at room temperature to visualize cell nuclei. After the final rinse, the specimens were mounted in fluorescence mounting medium (Dako Inc., Carpinteria, CA, USA). The specimens were analyzed using an FV1000 confocal microscope coupled to an IX81 inverted microscope (Olympus, Hamburg, Germany). Images were acquired at excitation wavelengths of 488 nm for FITC and 405 nm for DAPI. Analyses were performed under three experimental conditions: control, nutrient deficiency, and hypoxia. Quantitative fluorescence analysis was performed on 14 confocal images: five control images, four hypoxic images, and five nutrient-deficient images. For each image, fluorescence intensity histograms were used to calculate the total FITC fluorescence intensity corresponding to PGR and the total DAPI fluorescence intensity. Total fluorescence intensity was calculated as the sum of fluorescence intensity values multiplied by the corresponding number of pixels. PGR fluorescence was then normalized to DAPI fluorescence from the same image, and the results were expressed as the PGR/DAPI fluorescence ratio.

### 4.9. Statistical Analysis

Statistical analysis was performed on the results of relative *PGR* gene expression and PGR protein levels determined by ELISA, and normalized PGR/DAPI fluorescence ratios obtained from confocal microscopy analysis. Analyses were conducted using Statistica 13 software (StatSoft Polska, Kraków, Poland). Because two study models were used, namely a cell model and patient-derived tissue material, analyses were performed separately for each model. Data normality was assessed using the Shapiro–Wilk test. For data obtained from the cell model, a normal distribution was found in all analyzed groups; therefore, Student’s t test was used to compare the experimental conditions with the control group. For data obtained from GBM tissues, the distribution of variables was non-normal in most cases; therefore, the Mann–Whitney U test was used to compare analogous tumor regions between women and men, and the Wilcoxon signed-rank test was used to compare the analyzed tumor regions. Values of *p* < 0.05 were considered statistically significant.

## 5. Conclusions

The tumor microenvironmental conditions modeled in U87 cells increased *PGR* mRNA expression under hypoxic conditions but had no effect under nutrient deprivation. Neither condition altered PGR protein expression.

The individual regions of GBM showed different levels of PGR protein expression. In GBM, ***PGR*** mRNA expression did not correspond to PGR protein expression. No significant effect of sex onPGR expression in the individual regions of GBM.

PGR is present in GBM, and some tumor microenvironmental conditions affect its expression. A useful complement to the presented results would be to assess the activity of the receptor under investigation and the effects of its activation on cellular biological responses under different tumor microenvironmental conditions. This would allow a better understanding of its potential role in the biological processes associated with specific regions of GBM.

## Data Availability

The raw data supporting the conclusions of this article are available from the corresponding author upon reasonable request.
